# Beyond Resistance: Phenotypic Plasticity in Bacterial Responses to Antibiotics, Oxidative Stress and Antimicrobial Photodynamic Inactivation

**DOI:** 10.3390/molecules31030567

**Published:** 2026-02-06

**Authors:** Aleksandra Rapacka-Zdonczyk

**Affiliations:** Laboratory of Photobiology and Molecular Diagnostics, Intercollegiate Faculty of Biotechnology, University of Gdansk and Medical University of Gdansk, Abrahama 58, 80-307 Gdansk, Poland; aleksandra.rapacka-zdonczyk@ug.edu.pl

**Keywords:** antimicrobial resistance (AMR), antimicrobial blue light (aBL), antimicrobial photodynamic inactivation (aPDI), biofilm, oxidative stress, phenotypic plasticity, resilience, tolerance, persistence

## Abstract

The global challenge of antimicrobial resistance (AMR) has been framed primarily in terms of genetic resistance mechanisms. Nevertheless, bacteria can also survive antimicrobial stress through phenotypic plasticity, resulting in transient, non-genetic states such as tolerance, persistence, and population-level resilience. These phenotypic states complicate diagnostic efforts, diminish antibiotic efficacy, and contribute to the chronic nature of infections even in the absence of heritable resistance. This review evaluates phenotypic plasticity as a significant yet underrecognized factor in AMR, with a focus on responses to oxidative and photodynamic stress. Key manifestations of plasticity are discussed, including morphological and metabolic remodeling such as filamentation, small-colony variants, and metabolic rewiring, as well as envelope- and biofilm-associated heterogeneity and regulatory flexibility mediated by gene networks and horizontal regulatory transfer. The review highlights plastic responses elicited by reactive oxygen species-mediated stress and antimicrobial photodynamic inactivation, where single-cell heterogeneity, biofilm and mucus barriers, and light-dependent cues influence bacterial survival. Case studies are presented to demonstrate how photodynamic strategies can induce transient protective states and act synergistically with antibiotics, revealing mechanisms of action that extend beyond conventional single-target therapeutic models. Drawing on evidence from single-cell analyses, biofilm ecology, and experimental evolution, this review establishes phenotypic plasticity as a central element in the chemical biology of AMR. Enhanced understanding of plasticity is essential for advancing diagnostics, informing the development of adjuvant therapies, and predicting bacterial responses to novel antimicrobial interventions.

## 1. Introduction

Antimicrobial resistance (AMR) remains one of the most urgent threats to global health and has traditionally been explained by the acquisition of genetic determinants that confer resistance through drug inactivation, target modification, or efflux [1,2,3]. However, both clinical and laboratory observations increasingly demonstrate that bacteria can also survive lethal antimicrobial treatments in the absence of heritable resistance mutations [4,5]. This apparent paradox has shifted attention toward phenotypic plasticity and related non-genetic survival strategies, including tolerance, persistence, and resilience. Together, these transient states allow genetically susceptible bacteria to survive antimicrobial exposure and transiently mimic resistance phenotypes [6,7,8].

Phenotypic plasticity refers to the capacity of a single genotype to express multiple phenotypes in response to environmental cues and provides a unifying framework for understanding non-genetic bacterial adaptation. In bacteria, plasticity manifests across multiple biological scales. At the cellular level, it includes morphological alterations such as filamentation or the emergence of small-colony variants (SCVs) [9,10,11,12]. At the metabolic level, plastic responses involve pathway reprogramming that enhances defenses against oxidative stress [13,14,15]. At the population level, plasticity is reflected in biofilm-associated heterogeneity, which promotes tolerance and persistence through spatial organization and microenvironmental gradients [16,17,18,19]. While tolerance and persistence primarily reduce killing during antimicrobial exposure, resilience describes the capacity of bacterial populations to recover once stress is removed [20,21,22]. Together, these phenotypes form a continuum that blurs the classical boundary between susceptibility and resistance. The terms are contrasted in Table 1.

Reactive oxygen species (ROS) occupy a central position within this continuum. Several classes of conventional antibiotics induce oxidative stress or ROS accumulation as part of their downstream antimicrobial effects. Similarly, light-based antimicrobial strategies, including antimicrobial blue light (aBL) and antimicrobial photodynamic inactivation (aPDI), exert their activity predominantly through ROS-mediated damage [28,29,30,31]. Importantly, stress responses under these conditions are not uniform. In biofilms, phenotypic heterogeneity is shaped by biofilm-associated microenvironments, while oxidative and photodynamic stress further reveal pronounced heterogeneity at the level of individual cells [19,32,33].

Together, these observations underscore that AMR cannot be reduced to a single mechanism or a single adaptive trajectory. Phenotypic plasticity encompasses cue-dependent and reversible adaptations, such as filamentation, biofilm formation, and metabolic shifts, whereas bet-hedging generates stochastic, cue-independent variants, including persister cells [34,35,36,37,38].

Although oxidative stress is a common downstream component of many antimicrobial interventions, photodynamic stress represents a distinct and particularly informative framework for studying bacterial phenotypic plasticity. aPDI and aBL generate ROS through photochemical reactions, leading to multi-target damage affecting membranes, proteins, and nucleic acids simultaneously [39]. Unlike antibiotics, whose ROS-associated effects are often indirect, context dependent, or secondary to primary targets [40,41], photodynamic stress enables externally controlled ROS exposure with spatial and temporal modulation determined by light delivery parameters [29]. This controllability, together with the absence of a single dominant molecular target [30], makes aPDI uniquely suited to reveal non-genetic, heterogeneous bacterial responses and to dissect tolerance, persistence, and resilience as plastic outcomes rather than resistance-driven phenomena. For these reasons, photodynamic stress is treated throughout this review not as a specialized application, but as a complementary and experimentally powerful axis for examining bacterial phenotypic plasticity under oxidative stress.

This review first introduces conceptual frameworks of bacterial phenotypic plasticity, then surveys its major cellular and regulatory manifestations under oxidative and photodynamic stress, and finally discusses the implications of plasticity for antimicrobial therapy and bacterial evolvability.

**Figure 1:**
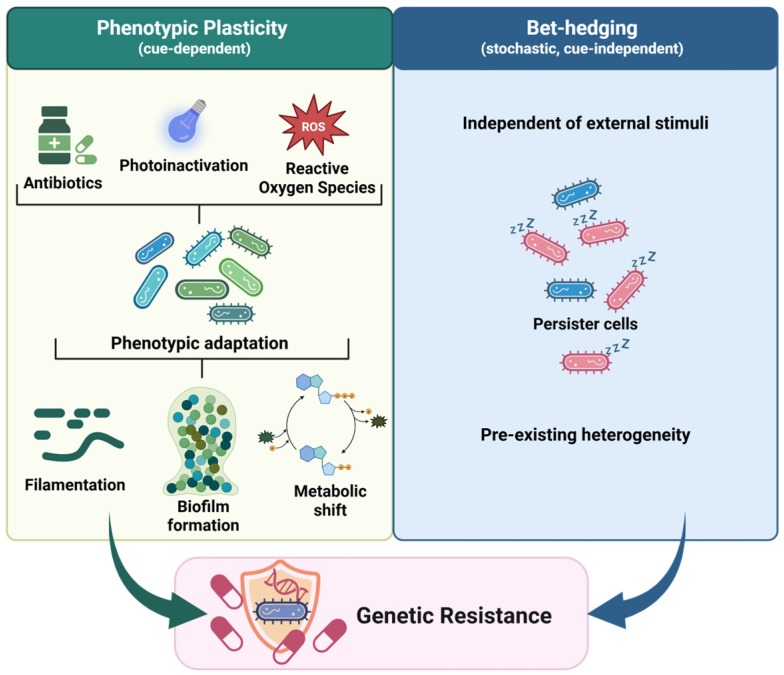
Bacterial survival strategies beyond classical genetic resistance. Phenotypic plasticity involves cue-dependent, reversible adaptations (e.g., filamentation, biofilm formation, metabolic shifts), whereas bet-hedging generates stochastic, cue-independent variants such as persister cells. External cues, including antibiotics, reactive oxygen species (ROS), or photoinactivation, can induce phenotypic plasticity, while bet-hedging arises independently of such stimuli. Both strategies are non-heritable and distinct from genetic resistance, yet can promote bacterial survival during antimicrobial exposure and thereby facilitate the eventual emergence of genetic resistance.

Figure 1 provides a conceptual overview of bacterial survival strategies beyond genetic resistance, contrasting cue-dependent plasticity with cue-independent bet-hedging and positioning both within a unified framework of non-genetic adaptation.

## 2. Conceptual Frameworks of Phenotypic Plasticity

This section introduces conceptual frameworks that help interpret phenotypic plasticity as a dynamic and context-dependent survival strategy in bacteria.

Phenotypic plasticity, defined as the capacity of a single genotype to produce multiple phenotypes in response to environmental variation, provides a useful conceptual framework for interpreting non-genetic bacterial survival under antimicrobial stress [25,27,42]. Although plasticity has been classically discussed in ecology and evolutionary biology [43,44,45], it is increasingly recognized as relevant to microbiology, where transient, non-heritable phenotypic states can substantially shape infection dynamics and responses to antimicrobial treatment [9,10,12].

### 2.1. Reaction Norms and Gene Regulatory Networks as Drivers of Plasticity

Phenotypic plasticity can be conceptualized through the framework of reaction norms, defined as the range of phenotypes that a single genotype can express across different environments. In bacteria, reaction norms extend beyond growth rates or morphological traits to encompass complex stress-associated phenotypes, including tolerance, persistence, and resilience. The breadth and shape of bacterial reaction norms reflect the capacity to cope with environmental variability and repeated stress exposure [26,27,46].

At the mechanistic level, reaction norms are underpinned by gene regulatory networks (GRNs) that integrate environmental signals with intracellular regulatory states. Central hubs such as sigma factors (e.g., RpoS, RpoE), two-component systems, and global regulators including (p)ppGpp coordinate large-scale transcriptional reprogramming in response to stress [47,48,49,50,51,52,53]. Through their connectivity and feedback structure, GRNs balance robustness, which stabilizes stress-responsive programs, with flexibility that permits alternative regulatory states [54]. Importantly, GRN-driven responses are frequently non-linear and context dependent. Identical stimuli may elicit distinct phenotypic outcomes depending on metabolic state, prior exposure (priming), or stochastic fluctuations in gene expression. Such combinatorial regulation can help explain how genetically identical bacterial populations can diverge into tolerant or persister subpopulations without genetic change, underscoring phenotypic plasticity as an emergent property of regulatory network dynamics [6,8,13,14,34,55,56,57,58,59,60,61].

### 2.2. Costs and Limits of Plasticity

Phenotypic plasticity is associated with both benefits and costs, which can be broadly divided into phenotypic costs and plasticity costs [25]. Phenotypic costs arise from trade-offs inherent to specific adaptive states; for example, filamentation may enhance survival under stress while simultaneously impairing cell division or proliferation. In contrast, plasticity costs reflect the energetic and regulatory demands associated with maintaining the molecular and regulatory architectures that enable flexible responses to environmental change [62]. Importantly, plasticity can sometimes mask underlying genetic variation by buffering populations from selective pressures, potentially slowing the rate of genetic adaptation [43,44]. Conversely, plasticity may contribute to faster evolutionary responses by revealing phenotypic diversity that becomes subject to selection following environmental change [45,63]. Together, these contrasting effects highlight plasticity primarily as a short-term survival strategy that can, under certain conditions, influence longer-term evolutionary trajectories.

Nevertheless, phenotypic plasticity has inherent limits. Reaction norms are typically shaped under moderate and relatively predictable environmental conditions. As a result, extreme stressors, such as intense oxidative bursts or photodynamic damage, may exceed the range over which plastic responses remain effective, thereby limiting the protective capacity of phenotypic adaptation [41,64].

### 2.3. Rate of Plasticity and Environmental Predictability

The success of plasticity depends not only on its scope but also on the timing of phenotypic responses relative to environmental change [43,44]. If environmental fluctuations occur faster than cells can adjust, plastic responses may fail to provide a benefit, limiting their adaptive value. Conversely, predictable cues can enable anticipatory responses. Light represents one such cue: in non-photosynthetic bacteria, it has been shown to trigger protective programs in anticipation of impending water loss, an ecologically coupled stress that may culminate in desiccation [65].

In pathogens such as *Acinetobacter baumannii*, the BLUF photoreceptor BlsA integrates light with temperature and metabolic status [66,67] to modulate iron acquisition [67,68], biofilm-associated traits [66,69], and oxidative stress-related responses [68,69].

Beyond BLUF systems, LOV and MerR/ChrR modules have been implicated in bacterial responses to blue-light-induced oxidative stress [70,71], while aBL has been reported to broadly alter bacterial physiology through multi-target, ROS-mediated damage affecting membranes, proteins, and nucleic acids [30,72,73].

In contrast, delayed activation of oxidative stress regulators such as OxyR can generate transient windows of vulnerability, characterized by elevated oxidative damage and mutagenesis [74,75]. Collectively, these observations indicate that the rate of plastic adjustment can influence whether bacterial populations predominantly survive transient stress, generate adaptive variation, or experience failure under adverse conditions.

### 2.4. Conceptual Links Between Plasticity and Evolutionary Rescue

Phenotypic plasticity has been proposed to contribute to evolutionary rescue, defined as the persistence of populations in deteriorating environments long enough for adaptive genetic change to occur [43,76]. By buffering immediate mortality and maintaining population size, plastic responses may increase the opportunity for genetic change, although this effect is strongly context dependent [20,21]. Experimental evolution studies further indicate that fluctuating environments tend to favor plastic phenotypes and generalist strategies, whereas constant environments promote specialization [44,77,78]. 

**Figure 2:**
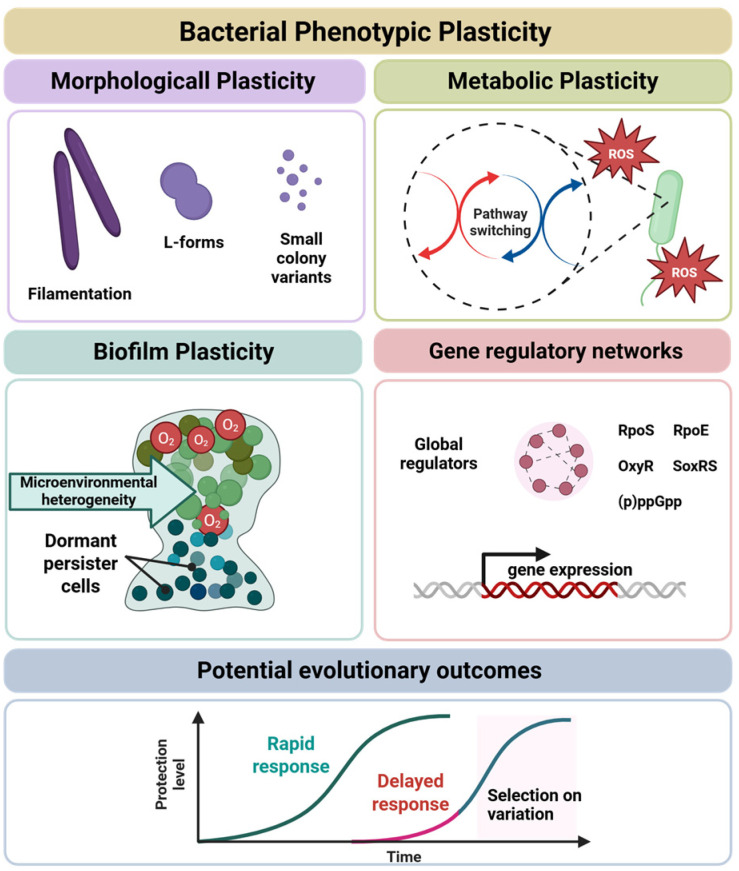
Conceptual frameworks and manifestations of bacterial phenotypic plasticity. Bacterial phenotypic plasticity manifests across multiple organizational levels. Morphological plasticity includes reversible changes such as filamentation, L-form transition, and the emergence of small-colony variants. Metabolic plasticity involves pathway switching and redox reprogramming that support tolerance to oxidative stress. Biofilm-associated plasticity arises from microenvironmental heterogeneity, leading to phenotypic diversification and the formation of dormant persister subpopulations. At the regulatory level, plastic responses are coordinated by gene regulatory networks governed by global stress regulators (e.g., RpoS, RpoE, OxyR, SoxRS, and (p)ppGpp), which dynamically reshape gene expression without requiring genetic change. At the population level, the timing of phenotypic responses influences stress outcomes: rapid plastic responses enhance transient protection, whereas delayed responses can create windows of vulnerability in which pre-existing or stress-induced variation becomes subject to selection. Together, these layers illustrate how phenotypic plasticity supports short-term survival and, under specific conditions, may influence longer-term evolutionary trajectories without implying deterministic evolutionary rescue.

Together, these findings suggest that phenotypic plasticity can act as a short-term safeguard against extinction and under specific conditions influence longer-term evolutionary outcomes, thereby linking microbial physiology with evolutionary theory [45]. Figure 2 illustrates the major manifestations of bacterial phenotypic plasticity, metabolic and morphological remodeling, biofilm- and envelope-associated heterogeneity, and regulatory or mobile element-driven flexibility, all of which contribute to transient protection and can facilitate evolutionary rescue under stress.

## 3. Cellular Manifestations of Phenotypic Plasticity

This section reviews the principal cellular manifestations of phenotypic plasticity that facilitate bacterial survival under oxidative, photodynamic, and other variable stresses. In bacteria, phenotypic plasticity occurs at multiple organizational levels, including metabolic reprogramming, morphological adaptation, envelope remodeling, and biofilm-associated heterogeneity. Collectively, these cellular adaptations demonstrate how non-genetic flexibility enables microbial populations to persist in the face of diverse environmental challenges.

### 3.1. Metabolic Plasticity

Metabolic plasticity plays a central role in bacterial stress survival. Bacteria are capable of redirecting carbon flux into the pentose phosphate pathway, which increases NADPH production and enhances antioxidant capacity [40,79,80]. At the population level, exposure to sublethal concentrations of hydrogen peroxide (H_2_O_2_) can induce protective gene expression, thereby reducing subsequent cell death [74]. In contrast, abrupt oxidative stress may result in a transient adaptation delay, during which delayed OxyR activation leaves a subpopulation vulnerable and increases mutagenesis [75]. 

Metabolic transitions also contribute to the formation of persister cells. In *Escherichia coli*, diauxic shifts generate tolerant subpopulations via a (p)ppGpp-dependent mechanism involving RelA/SpoT and DksA, which modulate transcription and DNA topology [81]. These adaptations illustrate the trade-offs inherent to plasticity: increased tolerance is frequently associated with reduced growth and, in some cases, altered motility [82,83,84]. Overall, metabolic plasticity coordinates redox balance, response timing, and carbon flux adjustments, thereby shaping bacterial survival under fluctuating environmental conditions.

### 3.2. Morphological Plasticity

A prominent manifestation of bacterial phenotypic plasticity is the capacity to undergo significant and reversible morphological changes in response to environmental stress.

Filamentation, induced by DNA damage and activation of the SOS response, exemplifies this plasticity. In this state, RecA-dependent SulA inhibits cell division, resulting in elongated filaments that temporarily protect against oxidative bursts and phagocytosis during DNA repair [9,10]. Another well-characterized form is the emergence of SCVs, which have been extensively characterized in *Staphylococcus aureus*, particularly in the context of chronic infections [11]. SCVs are typically associated with metabolic and regulatory changes that alter energy metabolism, rather than a single conserved mechanism. In *Pseudomonas aeruginosa* isolated from cystic fibrosis (CF) lungs, SCVs constitute a recurrent adaptive phenotype that promotes biofilm formation, antimicrobial tolerance, and persistence during chronic infection [85]. Comparable phenotypic diversification occurs in *E. coli* as it adapts to macrophage environments, where SCVs and mucoid morphotypes rapidly emerge and enhance survival under immune pressure. In this context, SCVs represent transient, heritable phenotypic variants that confer an early fitness advantage during intracellular infection but are eventually outcompeted by other adaptive phenotypes [86].

A further example is the transition into L-forms, or cell wall-deficient bacteria, which can be induced by β-lactam antibiotics and other stresses that interfere with cell wall integrity, including oxidative stress. These fragile yet proliferative forms are intrinsically tolerant to cell wall-targeting antibiotics and can propagate under osmotically protective conditions [87,88,89]. Finally, persisters often display distinct morphological correlates, typically smaller or metabolically inactive cells, representing a bet-hedging strategy embedded in structural heterogeneity [8,23].

Taken together, these examples, filamentation, SCVs, L-forms, and persister cells, illustrate how transient remodeling of bacterial architecture can promote survival through predominantly non-genetic mechanisms, thereby influencing infection dynamics and stress outcomes in both environmental and clinical contexts.

Recent work has begun to elucidate how dormancy and persistence are executed at the molecular and biophysical level, revealing a key role for biomolecular condensates formed via liquid–liquid phase separation (LLPS). In bacteria, diverse stress conditions induce the reversible assembly of phase-separated, membraneless condensates that reorganize cytoplasmic processes without requiring genetic change [90,91,92,93,94]. These condensates function as regulatory hubs by sequestering components of the translational machinery and stress-associated regulators, thereby enforcing growth arrest and metabolic downshifts characteristic of dormant and persister states [90,91,94,95]. Importantly, condensate formation is dynamic and tunable: liquid-like assemblies can mature toward more solid-like aggregates during prolonged stress, a transition associated with deeper dormancy and reduced recovery potential [96]. Single-cell analyses and evolutionary observations suggest that LLPS-mediated sequestration of amino-acyl-tRNA synthetases and translation-associated factors can coincide with reduced translational activity and the appearance of dormancy-like phenotypes in a subpopulations of otherwise growing cells [94,95]. Collectively, these findings provide a biophysical mechanism linking stress sensing, morphological plasticity, and non-genetic survival, positioning phase separation as a fast, reversible switch that enables bacterial populations to execute dormancy as part of plastic and bet-hedging strategies rather than through genetically fixed programs.

### 3.3. Envelope and Biofilm Plasticity

The envelope is a frontline of stress adaptation. Regulation of porins, efflux pumps, and envelope stress responses (e.g., RpoS) buffers oxidative challenges while modulating antibiotic tolerance [41,97,98].

At the community scale, biofilms embody plasticity. Oxygen, nutrient, and ROS gradients generate subpopulations with distinct phenotypes, including persisters [16,33,98]. Transcriptomic and proteomic analyses compiled across multiple studies show that biofilm growth alters expression of large genomic fractions, enriching stress defense and metabolic pathways [32].

The biofilm matrix (extracellular polymeric substances, EPS) creates dynamic diffusion barriers and microgradients, enabling cooperative defense and localized niches [17,99,100]. ROS responses are emergent, collective properties of bacterial communities, shaped by local context and population heterogeneity rather than uniform single-cell behavior [33].

Biofilms also harbor viable but non-culturable (VBNC) cells, a debated but recognized dormancy form [101,102,103]. VBNC cells retain viability markers such as membrane integrity but are unable to grow on standard culture media, resuscitating only under permissive conditions. The relationship between VBNC cells and persister cells, whether they represent distinct, overlapping, or continuous dormancy states, remains actively debated [104,105,106].

From an evolutionary perspective, fluctuating environments favor phenotypically plastic biofilm producers, whereas stable, static conditions select for specialist non-producers or constitutive strategies [107]. Thus, biofilms represent hotspots of phenotypic plasticity, integrating envelope regulation, metabolic rewiring, and dormancy into collective modes of survival [108].

### 3.4. Regulatory Plasticity

Regulatory plasticity arises from the dynamic organization of gene regulatory networks, providing flexibility that extends beyond changes in protein-coding sequences. In *Bacillus subtilis*, Rap/Phr phosphatases and their cognate peptides exemplify such plastic regulation by fine-tuning entry into sporulation, competence, and biofilm-associated states in a context-dependent and population-coordinated manner [109].

Additional layers of regulatory plasticity can arise through horizontal regulatory transfer (HRT), in which noncoding DNA elements, such as promoters or regulatory regions, are mobilized across genomes. These events can rewire transcriptional networks without altering protein-coding sequences, thereby enabling novel regulatory phenotypes [110].

Together with redundancy in gene regulatory networks [111] and epigenetic memory mechanisms, including phase variation and DNA methylation [112], these processes expand the range of phenotypic states accessible to bacteria. Importantly, they illustrate how regulatory architecture itself can act as a substrate for phenotypic diversification and flexible stress responses without requiring immediate genetic change.

### 3.5. Mobile Genetic Elements and Plasticity

Although mobile genetic elements (MGEs) involve heritable genetic material, their immediate effects on bacterial physiology are often regulatory rather than mutational.

Through gene dosage, regulatory interactions, and context-dependent expression, MGEs can blur the boundary between genetic change and phenotypic plasticity during short-term stress responses.

MGEs, including plasmids, integrative and conjugative elements (ICEs), and bacteriophages, are traditionally viewed as vectors of horizontal gene transfer (HGT). However, they can also act as powerful modulators of phenotypic plasticity by reshaping regulatory architectures and physiological states independently of stable genetic fixation. Plasmids in particular exemplify this dual role. Beyond transmitting resistance determinants, plasmids directly influence bacterial fitness through regulatory interactions, metabolic burdens, and environment-dependent trade-offs. For instance, plasmid-bearing strains often exhibit reduced growth rates yet enhanced survival under stress, reflecting a context-dependent balance between metabolic costs and stress-adaptive benefits in fluctuating environments [113].

Importantly, plasmids do not function solely as carriers of protein-coding genes. Many also encode noncoding regulatory loci or toxin–antitoxin modules that can rewire host transcriptional programs and expand the spectrum of accessible phenotypic states [114]. These interactions are inherently coevolutionary, as donors and recipients reciprocally shape one another’s physiology rather than engaging in unidirectional gene transfer [115]. Clinically, plasmid-mediated tolerance and persistence can stabilize or reverse depending on treatment regimens, highlighting the role of MGEs in shaping dynamic, context-dependent responses to antimicrobial stress [116].

At the community level, plasmids also influence microbiome stability and ecosystem function, positioning them as important contributors within a One Health framework [117,118]. ICEs and bacteriophages further broaden this phenotypic flexibility by mobilizing regulatory modules and stress-response genes, enhancing adaptability under fluctuating environmental conditions [119]. In this context, MGEs should be recognized not only as vehicles of resistance determinants but also as amplifiers of bacterial phenotypic plasticity, coupling HGT with regulatory rewiring and shaping both short-term tolerance and longer-term evolutionary trajectories. 

**Figure 3:**
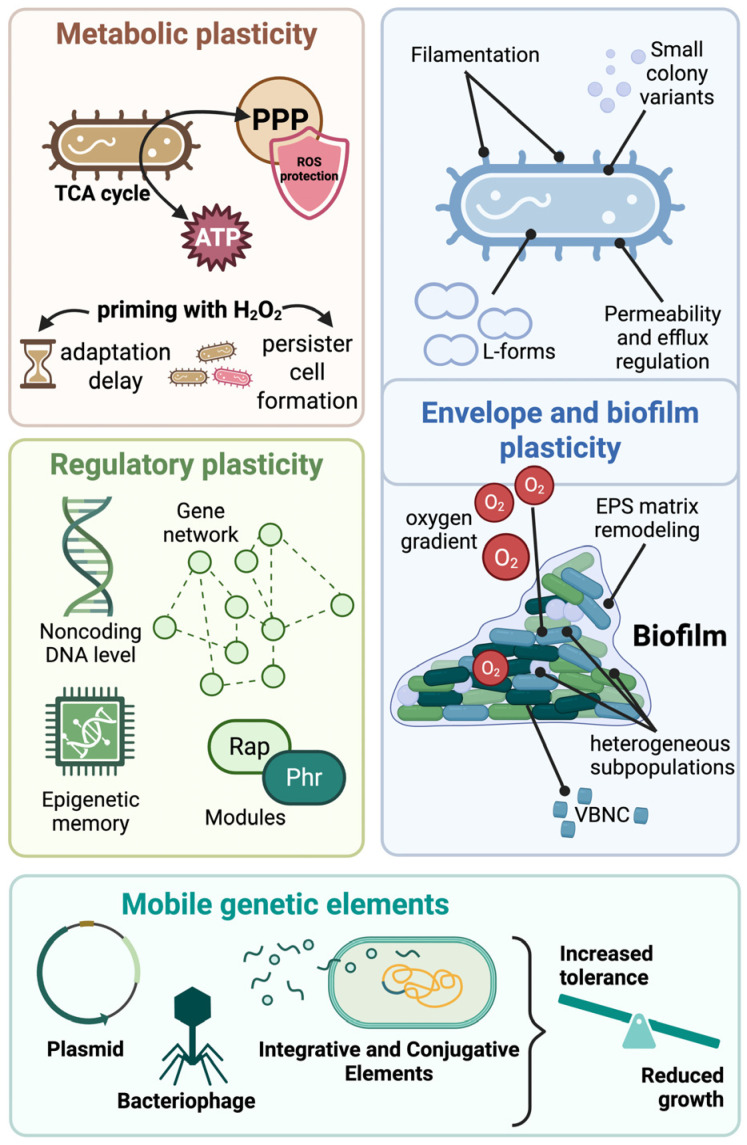
Cellular manifestations of bacterial phenotypic plasticity. Bacteria deploy multiple, interconnected layers of phenotypic plasticity to survive fluctuating environments. Metabolic plasticity encompasses carbon flux rerouting (e.g., PPP versus TCA cycle), antioxidant defenses, priming responses, and timing-dependent effects such as adaptation delay or persister formation. Morphological and envelope plasticity include filamentation, small colony variants (SCVs), L-forms, and regulation of envelope permeability and efflux systems. Biofilm plasticity reflects the emergence of heterogeneous subpopulations shaped by spatial organization and physicochemical gradients. These include oxygen limitation, EPS matrix remodeling, and the transient entry of cells into persister or VBNC states. Regulatory plasticity arises from redundant gene networks, Rap/Phr signaling modules, epigenetic memory, and horizontal transfer of regulatory noncoding DNA. Mobile genetic elements (plasmids, ICEs, and bacteriophages) further extend phenotypic plasticity by coupling horizontal gene transfer with regulatory rewiring, generating context-dependent trade-offs between growth, tolerance, and survival.

The major cellular and regulatory manifestations of bacterial phenotypic plasticity discussed in this section are summarized in Figure 3.

## 4. Plasticity Under Oxidative and Photodynamic Stress

Phenotypic plasticity is especially evident under oxidative stress, where bacterial survival depends not only on tolerance to ROS-mediated damage but also on the capacity to sense and respond to specific redox perturbations. ROS differ in their reactivity, diffusion ranges, and molecular targets, leading to selective modulation of specific cellular pathways rather than causing uniform, nonspecific damage [120,121].

For example, H_2_O_2_ preferentially oxidizes redox-sensitive cysteine residues and activates transcriptional regulators such as OxyR, which coordinate antioxidant defenses and metabolic reprogramming. In contrast, superoxide predominantly disrupts enzymes and regulators containing iron–sulfur clusters, thereby altering central metabolism and respiratory pathways [120,122,123].

Notably, ROS-mediated modifications extend beyond compromising macromolecular integrity. They can actively reconfigure regulatory networks that control DNA repair, stress responses, and metabolic fluxes. Consequently, antimicrobial strategies that generate ROS often yield heterogeneous survival outcomes, including tolerance, persistence, and altered recovery dynamics, rather than uniform bactericidal effects [120,121].

Within this framework, aPDI and aBL are particularly well suited to probe plastic responses, as they generate specific ROS in situ via photochemical reactions and allow for spatially and temporally controlled redox stress [124]. Collectively, these approaches elucidate plastic responses at both single-cell and community levels, thereby connecting oxidative stress adaptation with tolerance, persistence, and therapeutic outcomes.

### 4.1. Regulatory Stress Networks Engaged by Photodynamic Versus Oxidative Stress

Photodynamic stress activates several canonical bacterial stress response pathways that are also engaged during general oxidative stress, including redox-sensitive regulators such as OxyR, DNA damage-associated SOS responses, and global stress programs related to envelope and stationary-phase regulation [29,125,126]. However, the mode, coordination, and population-level synchrony of these responses differ significantly from those triggered by uniform oxidants, such as exogenous H_2_O_2_, which is often used as a simplified oxidative stress model, or by antibiotic-induced oxidative stress, where ROS generation is typically indirect and context-dependent [120,122,127,128,129]. While H_2_O_2_ generally induces a coordinated, OxyR-dependent transcriptional response across the population, photodynamic stress results in spatially and temporally heterogeneous ROS exposure. Consequently, stress regulators are activated asynchronously and cell-specifically, leading to divergent survival outcomes [120,122,126].

In addition to redox-responsive pathways, photodynamic stress frequently intersects with broader global stress responses. DNA damage arising from singlet oxygen and secondary ROS promotes activation of the SOS response [28,130]. Concurrent protein oxidation and membrane perturbation are hallmark consequences of photodynamic stress. These conditions are consistent with engagement of RpoS-mediated general stress programs, rather than a dedicated photodynamic signaling pathway [48]. Although direct induction of the stringent response by photodynamic stress has not been conclusively demonstrated, the alarmone (p)ppGpp is widely recognized as a central integrator of diverse stress signals (including nutrient limitation, translational perturbations, and oxidative challenges) and plays a key role in tolerance and persistence phenotypes. Given that photodynamic stress perturbs multiple cellular processes known to converge on stringent-like regulatory pathways, (p)ppGpp-mediated signaling represents a plausible, albeit indirect, contributor to plastic survival responses under light-based antimicrobial stress.

Regulatory responses to oxidative and photodynamic stress are not uniformly synchronized across bacterial populations, as evidenced by single-cell analyses that reveal heterogeneous stress engagement and survival outcomes [33,131]. Consequently, photodynamic stress creates a distributed and heterogeneous regulatory environment, making it particularly suitable for investigating phenotypic plasticity, tolerance, and recovery dynamics.

### 4.2. Single-Cell Heterogeneity in Photodynamic Inactivation

aPDI may not eliminate bacterial populations uniformly. Even genetically identical cells exhibit markedly heterogeneous outcomes, reflecting non-genetic variability in photosensitizer interactions with the cell envelope, ROS-mediated damage, and stress response dynamics. Time-resolved single-cell microscopy has demonstrated that individual bacteria can vary significantly in survival times, ranging from seconds to over an hour, with these differences best explained by phenotypic plasticity rather than stable resistance mutations [131]. Notably, no surviving subpopulation was detected after treatment, highlighting the transient and reversible nature of this phenotypic heterogeneity.

An independent study on *Streptococcus agalactiae* showed that repeated sublethal Rose Bengal-mediated aPDI promoted the emergence of SCVs, altered hemolytic activity, and induced additional phenotypic changes, suggesting that iterative low-level exposure can enhance tolerance through heterogeneous, non-genetic responses [132]. However, this does not indicate inherent resistance of SCVs to light-based antimicrobials. Although SCVs are known to reduce antibiotic efficacy, experimental evidence indicates that *S. aureus* SCVs can be effectively inactivated by advanced nanoparticle-assisted aPDI, where high local ROS generation overcomes SCV-associated tolerance mechanisms [133].

Taken together, these findings illustrate the dual nature of photodynamic inactivation. On the one hand, it can provide a powerful means to eliminate SCVs that evade conventional antibiotics. On the other hand, repeated sublethal exposures may promote the emergence of transient tolerance phenotypes, complicating treatment outcomes.

### 4.3. Biofilm as a Plasticity Hotspot Under Oxidative and Photodynamic Stress

Under oxidative and photodynamic stress, biofilms serve as prime examples of community-level phenotypic plasticity. Spatial gradients inherent to biofilm architecture influence photosensitizer penetration, light distribution, and local ROS exposure, leading to heterogeneous killing and survival outcomes [134]. These ROS responses do not indicate intrinsic resistance at the single-cell level but instead arise as collective, context-dependent properties shaped by microenvironmental structure and intercellular interactions [33].

From a therapeutic perspective, this plasticity represents both a challenge and an opportunity. Multiple studies have shown that aPDI can disrupt biofilm resilience and act synergistically with antibiotics or host immune factors to eradicate biofilm-associated infections [135]. Combined aPDI-antibiotic and aPDI–host defense strategies have been demonstrated to enhance the clearance of *S. aureus* biofilms, underscoring the potential of photodynamic approaches to overcome biofilm-mediated tolerance [136]. Additionally, phenothiazine- and malachite green–based photosensitizers have been reported to significantly reduce biofilms formed by both *S. aureus* and *E. coli* [137]. More recently, aPDI has been shown to alter antibiotic susceptibility in *Enterococcus* spp., supporting the view that biofilms act as dynamic hubs of phenotypic plasticity and that photodynamic approaches may help counteract ROS-associated tolerance and persistence [138].

Collectively, these findings emphasize that community-level heterogeneity and spatial organization within biofilms influence stress outcomes under ROS-based interventions, generating both obstacles to eradication and opportunities for therapeutic exploitation.

### 4.4. Cross-Talk with Antibiotics

Phenotypic plasticity induced by oxidative and photodynamic stress has direct, context-dependent effects on antibiotic efficacy. ROS exposure can promote transient phenotypes such as filamentation or the emergence of SCVs, which are associated with reduced antibiotic susceptibility without stable genetic resistance [10,11]. These plastic responses may temporarily protect bacterial populations from antimicrobial killing, thereby complicating treatment outcomes.

In contrast, light-based therapies can sensitize bacteria to antibiotics by damaging resistance determinants and cellular structures. ROS-mediated inactivation of β-lactamases, disruption of membrane integrity, and increased intracellular stress have all been shown to enhance antibiotic activity [31,139]. Consistent with these mechanisms, synergistic effects between aPDI or aBL and antibiotics have been observed in various experimental and applied settings, including *S. aureus* skin infections [140], multidrug-resistant bacteria in hospital wastewater [141], and advanced nanoparticle-assisted photodynamic systems [142]. Mechanistic reviews further highlight the ability of ROS-centered therapies to enhance antibiotic efficacy rather than serve solely as standalone antimicrobials [39,143].

Importantly, the consequences of phenotypic plasticity differ across species. In *S. aureus*, survivors of phototreatment exhibited marked sensitization to some antibiotics, indicating that tolerance to aPDI may involve a fitness trade-off manifested as enhanced drug susceptibility [130]. In contrast, *E. coli* populations tolerant to aBL exhibited largely unchanged antibiotic susceptibility profiles, with only minor twofold MIC variations that did not affect clinical classification [144].

**Figure 4:**
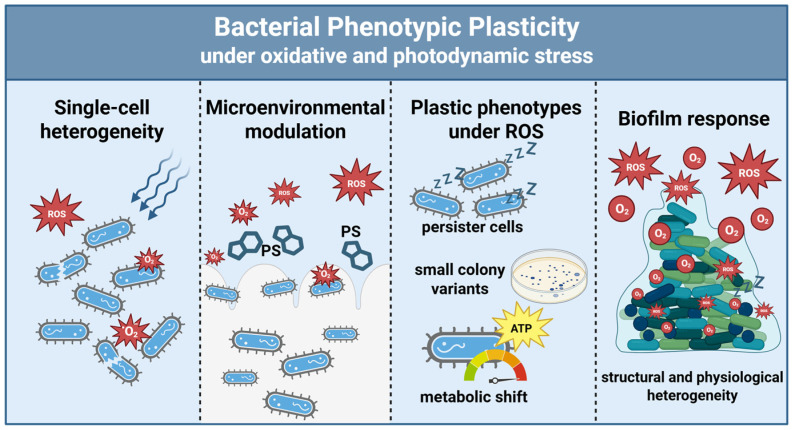
Bacterial phenotypic plasticity under oxidative and photodynamic stress. aBL and aPDI generate ROS, revealing the heterogeneous and context-dependent nature of bacterial stress responses. At the single-cell level, variability in photosensitizer uptake, ROS detoxification, and stress signaling leads to divergent survival outcomes. Microenvironmental factors, including mucus and biofilm matrix components, modulate ROS diffusion and photosensitizer activity. These conditions can give rise to plastic phenotypes such as metabolic rewiring toward redox balance (e.g., increased NADPH production), persister cells, and SCVs, which confer transient stress tolerance. Within biofilms, spatial ROS gradients and structural heterogeneity may promote tolerant and persistent subpopulations, positioning biofilms as clinically relevant hotspots of phenotypic plasticity.

These divergent outcomes underscore the complexity of phenotypic plasticity. While plastic responses can hinder therapeutic predictability, they may also create context-dependent vulnerabilities that can be leveraged through synergistic interventions. Collectively, these findings establish oxidative and photodynamic stress as valuable experimental frameworks for investigating bacterial phenotypic plasticity at both cellular and community levels (Figure 4).

## 5. From Plasticity to Evolvability

This section explores the role of phenotypic plasticity in linking immediate stress survival to long-term evolutionary outcomes in bacterial populations. Phenotypic plasticity influences both short-term survival and the evolutionary trajectories of microbial populations under stress. Through mechanisms such as damage buffering, exposure of cryptic variation, and modulation of mutation supply, plastic responses may either constrain or accelerate adaptation.

In addition to cue-dependent plasticity, bet-hedging serves as a complementary strategy whereby stochastic diversification produces pre-adapted subpopulations in the absence of environmental cues. Collectively, these mechanisms link microbial stress physiology in oxidative and photodynamic environments to broader evolutionary concepts, such as plasticity-first dynamics, evolutionary rescue, and the maintenance of phenotypic heterogeneity as a substrate for adaptation.

### 5.1. Plasticity-First and the Baldwin Effect

The Baldwin effect is a theoretical and conceptual framework, rather than a universally established evolutionary mechanism, that posits that phenotypic plasticity may precede and influence subsequent genetic adaptation. According to this framework, transient, stress-induced traits can initially enhance survival and, under sustained selection, may become genetically assimilated [26,145,146,147]. In bacteria, this perspective implies that phenotypes such as filamentation, SCVs, or biofilm-associated tolerance may arise through non-genetic mechanisms, confer short-term survival benefits, and potentially shape longer-term evolutionary trajectories.

It is important to note that the Baldwin effect is presented here as a conceptual and heuristic framework, not as a demonstrated causal mechanism of genetic assimilation in bacteria. Direct evidence for Baldwin-type dynamics under photodynamic stress is limited. Nevertheless, several observations align with a plasticity-first scenario. In *Streptococcus pyogenes*, repeated sublethal cycles of curcumin-mediated aPDI led to altered biofilm formation and a gradual decrease in photosensitizer uptake, without the emergence of a stable resistance phenotype [148]. In *S. aureus*, survival after aPDI was primarily linked to transcriptional stress responses indicative of phenotypic plasticity. Repeated sublethal exposures were associated with rare regulatory mutations, including in *qsrR*, suggesting that prolonged photodynamic stress may foster conditions favorable for genetic adaptation following an initial plastic phase [149]. Similarly, in *S. agalactiae*, consecutive sublethal rose Bengal-aPDI treatments promoted phenotypic diversification, such as SCV-like colonies, altered hemolytic activity, and transcriptomic remodeling, again indicating tolerance mediated mainly by non-genetic mechanisms [132]. Notably, *S. aureus* populations tolerant to aPDI or aBL accumulated higher frequencies of rifampicin-resistant mutants over time, supporting the hypothesis that stress-induced plasticity may create transient windows for subsequent mutational change [130].

Overall, these findings do not establish a causal mechanism for genetic assimilation or demonstrate that plastic traits become genetically fixed. Nonetheless, they are consistent with the Baldwin-effect framework, wherein stress-induced phenotypic plasticity may link short-term resilience to the eventual emergence of heritable adaptations.

### 5.2. Bet-Hedging as a Complementary Strategy to Plasticity

Phenotypic plasticity is typically defined as a cue-dependent process in which cells detect environmental signals and adjust their phenotype accordingly. In contrast, bet-hedging is cue-independent; populations stochastically diversify phenotypes before stress occurs, thereby reducing the risk of catastrophic loss in unpredictable environments. Plasticity enhances fitness in relatively predictable conditions, whereas bet-hedging promotes survival under uncertainty by maintaining subpopulations that are pre-adapted to stress [34,36,38,150]. Phase variation, the reversible switching of surface structures, exemplifies this strategy and illustrates how stochastic diversification can increase population robustness [151]. Although bet-hedging increases survival under uncertainty, it does not inherently lead to directional evolutionary change or genetic assimilation.

Canonical examples of bet-hedging in bacteria include spontaneous formation of persister cells, which occurs independently of external cues, and stochastic activation of toxin–antitoxin modules that partition populations into metabolically active and dormant subgroups [6,53,152]. Biofilm-associated heterogeneity arises from the combined effects of environmental gradients and stochastic diversification, leading to subpopulations with increased tolerance to antibiotics or oxidative stress [34,35,37].

In clinical contexts, phenotypic plasticity and bet-hedging frequently coexist. For instance, in the lungs of CF patients, *P. aeruginosa* displays extensive phenotypic diversification, including plastic metabolic rewiring under hypoxic and biofilm-associated conditions, as well as the emergence of SCVs and subpopulations enriched in persister cells [85,153].

Collectively, these strategies contribute to the long-term persistence of bacterial populations and diminished efficacy of antimicrobial treatments.

### 5.3. Burst Mutagenesis Under Oxidative Stress

The timing of stress responses is critical in modulating bacterial evolvability. Lagage et al. (2023) [75] showed that delayed activation of the OxyR regulon under oxidative stress creates a transient period of elevated mutagenesis, referred to as an “adaptation delay”. During this period, unrepaired oxidative DNA lesions accumulate, temporarily increasing the mutation supply [75]. In contrast, priming with sublethal ROS exposures induces protective transcriptional programs that enhance stress tolerance, suppress mutagenesis, and stabilize phenotypes [74].

Thus, variations in the timing of plastic stress responses can influence evolutionary trajectories by altering the mutation supply, potentially promoting genetic diversification under delayed responses or buffering genetic change when protective programs are activated [154,155,156]. These findings indicate that phenotypic plasticity can shape evolutionary dynamics in opposing ways: maladapted or delayed responses may accelerate genetic diversification by increasing mutation rates, whereas timely activation of protective programs can buffer genetic change by limiting DNA damage. It is important to note that these effects reflect modulation of mutation supply and do not demonstrate that transient increases in mutagenesis result in stable adaptive genetic change.

### 5.4. Generalists via Phenotypic Switching

Phenotypic plasticity can facilitate generalist strategies in fluctuating environments by enabling reversible switching between alternative phenotypic states. Experimental evolution studies demonstrate that temporally variable conditions favor plastic phenotypes capable of exploiting multiple ecological niches, whereas constant environments select for specialists optimized for a single strategy [44].

Experimental evolution studies in Pseudomonas fluorescens have shown that trade-offs between traits such as growth and surface-associated phenotypes can be mitigated through stochastic phenotypic switching, enabling persistence across heterogeneous and fluctuating environments [157]. Similar switching between planktonic and biofilm-associated states has been reported in *Vibrio cholerae*, where it confers fitness advantages under environmental turnover [107].

Collectively, these observations support the conclusion that phenotypic plasticity enhances ecological versatility, offering a foundation for persistence and adaptation in diverse host and environmental contexts.

### 5.5. Limits of Plasticity in Extreme Environments

Despite its advantages, phenotypic plasticity has inherent limitations. Reaction norms typically evolve under moderate and recurrent environmental conditions; therefore, extreme stresses, such as high-dose oxidative bursts or intense photodynamic exposure, may exceed the range in which plastic responses are effective and can ultimately lead to population collapse. At the metabolic level, constraints imposed by multifunctional enzymes and essential pathways limit the extent to which central metabolism can be reconfigured without compromising viability [62]. These constraints align with observations that aBL and aPDI remain effective in many settings, as they impose stress regimes that may surpass the capacity of phenotypic flexibility.

The antibacterial action of aBL involves the simultaneous induction of multiple oxidative stress components, which cannot be fully replicated by exposing bacteria to individual stressors alone. Kruszewska-Naczk et al. (2024) demonstrated that single stressors only partially reproduce the biological effects of aBL [129]. Moreover, despite extensive experimental investigations across various bacterial species, treatment protocols, and methodologies, no stable, heritable resistance to aPDI or aBL has been convincingly documented to date [134]. While this does not preclude future evolutionary adaptation, it supports the existence of fundamental constraints on both plastic and genetic responses to ROS-based light therapies [158].

### 5.6. Plasticity and Evolutionary Rescue

Phenotypic plasticity is central to evolutionary rescue, defined as the capacity of populations to persist under deteriorating conditions long enough for adaptive processes to occur. Conceptual models suggest that plastic responses can buffer mortality and reduce extinction risk, thereby providing time for evolutionary change [159].

Experimental evidence supports this framework in microbial systems. In *P. aeruginosa*, eco-evolutionary dynamics under oxidative stress imposed by host immune defenses revealed that phenotypic switching alters adaptation trajectories and promotes persistence in hostile environments [63]. Similarly, microbial evolution experiments indicate that fluctuating environments favor plastic phenotypes, which delay extinction and increase the likelihood of subsequent resistance emergence [44,154].

Bet-hedging strategies can also provide a complementary rescue function by ensuring the presence of pre-adapted subpopulations, independent of environmental cues. Together, phenotypic plasticity and bet-hedging connect microbial stress physiology to evolvability, enabling immediate survival under stress and shaping the conditions for the potential emergence of heritable resistance.

**Figure 5:**
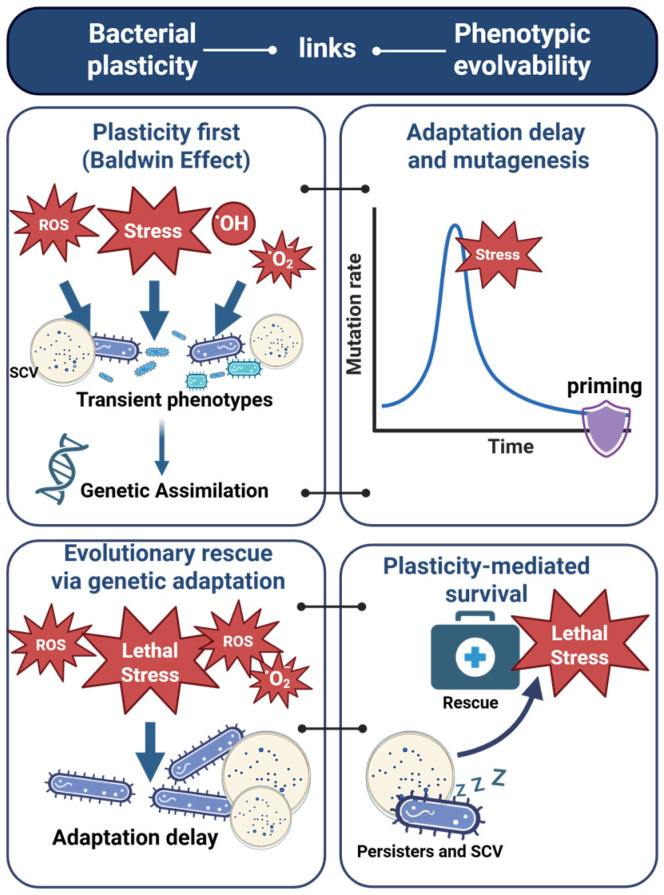
Links between bacterial phenotypic plasticity and evolvability under oxidative and photodynamic stress. Phenotypic plasticity can shape evolutionary trajectories through multiple, non-exclusive pathways. Stress-induced plastic responses (e.g., filamentation, SCVs, dormancy) may provide immediate survival benefits and, under sustained selection, *bias* subsequent genetic adaptation, consistent with a plasticity-first (Baldwin-effect) framework. In parallel, delayed or maladapted stress responses can create transient windows of elevated mutagenesis (“adaptation delay”), increasing genetic variation available for selection, whereas timely stress priming can buffer mutation rates. Plastic phenotypes such as persister cells can also promote population persistence under otherwise lethal stress, enabling evolutionary rescue when beneficial mutations arise and spread. Together, these pathways illustrate how phenotypic plasticity links short-term stress survival with longer-term evolvability, without implying deterministic genetic assimilation.

The principal forms of bacterial plasticity addressed in this review are summarized in Table 2. Figure 5 presents an integrative framework that links non-genetic stress responses to evolutionary outcomes.

## 6. Discussion and Future Directions

While phenotypic plasticity has long been recognized in ecology and evolutionary biology, its systematic integration into microbial pathogenesis and antimicrobial therapy remains limited. Moving forward, several conceptual and methodological advances will be required to more fully integrate plasticity into infection biology and antimicrobial therapy.

### 6.1. Standardizing Metrics of Plasticity

Current antimicrobial susceptibility testing is largely centered on minimum inhibitory concentration (MIC) values, which effectively capture heritable resistance but fail to adequately capture phenotypic tolerance, persistence, and resilience. As a result, clinically relevant non-genetic survival strategies often remain undetected. A key challenge for the field is therefore the development and standardization of complementary metrics, such as the minimum duration of killing (MDK), post-stress recovery kinetics, and single-cell survival assays, that can more accurately discriminate resistance from plasticity-driven survival [4,5].

Importantly, these metrics capture distinct dimensions of bacterial behavior under stress, including killing dynamics, heterogeneity of survival, and the capacity for post-treatment recovery, rather than growth inhibition alone. From a clinical perspective, plasticity-aware diagnostics do not necessarily require wholesale replacement of existing susceptibility testing workflows. MDK-based measurements and post-treatment recovery assays could be implemented as adjuncts to standard MIC testing in reference laboratories, particularly in the context of chronic, relapsing, or biofilm-associated infections. In such settings, these readouts may help identify tolerance-associated treatment failure and guide the rational use of combination or adjuvant therapies.

At present, MDK and recovery kinetics appear to represent the most immediately translatable metrics for near-term clinical application, whereas single-cell survival assays and high-dimensional imaging approaches remain primarily research tools. The latter provide critical mechanistic insight and enable stratification of plastic phenotypes, but their routine clinical implementation will require further simplification, standardization, and validation. Harmonizing plasticity-aware measurements across laboratories and clinical settings will be essential for integrating phenotypic plasticity into antimicrobial diagnostics and for moving beyond a resistance-centric framework that underestimates the contribution of non-genetic survival strategies to treatment failure.

### 6.2. Integrating Single-Cell and Systems Approaches

Recent advances in imaging, flow cytometry, and single-cell sequencing now allow phenotypic heterogeneity to be resolved at the level of individual bacterial cells, uncovering stress responses that remain hidden in bulk measurements [164,165]. In parallel, multi-omics and metabolic profiling approaches provide system-wide insights into stress-induced reprogramming and associated survival outcomes [166].

Integrating single-cell analyses with population- and systems-level approaches will be critical for understanding phenotypic plasticity across biological scales, from transient transcriptional rewiring in individual cells, to emergent properties such as biofilm resilience and population recovery dynamics [6,57]. Computational frameworks, including machine learning, are likely to enhance the classification and prediction of plastic phenotypes under diverse stress conditions.

### 6.3. Targeting Plasticity Therapeutically

Although phenotypic plasticity complicates treatment by promoting tolerant and persister states, it simultaneously reveals therapeutic vulnerabilities. aBL demonstrates this duality by inducing multi-target oxidative damage, which disrupts membranes and β-lactamases and synergizes with antibiotics rather than selecting for classical resistance [30,31,139]. Additionally, aPDI-induced hypoxia can activate prodrugs such as metronidazole [167]. In addition to direct bactericidal effects, plasticity-associated regulatory networks, including the SOS response and quorum-sensing systems, offer further intervention points that regulate entry into transient survival states [168,169]. Targeting these regulatory mechanisms may destabilize tolerant phenotypes before they develop into heritable resistance, thereby shifting therapeutic strategies from cell eradication to disruption of adaptive states.

Importantly, plasticity-aware interventions can also be implemented at the level of biomaterial interfaces, where early disruption of biofilm establishment may prevent the emergence of tolerant and persistent phenotypes. For example, nisin-enriched coatings on titanium implants were shown to effectively inhibit *S. aureus* biofilm formation and improve host survival in a *Galleria mellonella* implant infection model, illustrating a proactive anti-biofilm strategy that limits non-genetic survival without relying on systemic antibiotic pressure [170].

Complementary strategies may be developed to eradicate biofilm-resident persister subpopulations by employing synergistic combinations of physical and antimicrobial stressors. Verheul et al. demonstrated that non-contact induction heating (NCIH) in combination with the synthetic antimicrobial peptide SAAP-148 effectively eliminated persister cells within MRSA biofilms grown on metal surfaces, thereby simulating implant-associated infection. This study serves as a concrete example of a persister-targeting, plasticity-aware intervention, where the integration of a physical perturbation with a targeted antimicrobial agent disrupts tolerant survival states that are resistant to conventional antibiotic therapy [171]. Subsequently, this approach was applied to clinically relevant implant models, revealing that the timing and sequence of NCIH induction relative to antimicrobial exposure are critical determinants of biofilm eradication efficacy. These findings indicate that biofilm-associated tolerance is a dynamically regulated plastic state, rather than a fixed property, and can be therapeutically destabilized through appropriately timed, multimodal interventions [172].

### 6.4. Clinical and Translational Relevance

Phenotypic plasticity plays an important role in chronic infections, where biofilm heterogeneity, mucus barriers, and fluctuating host immune pressures promote tolerant and persistent phenotypes rather than stable genetic resistance [99,173]. Clinically, this is well illustrated by chronic *P. aeruginosa* infections in CF and ventilator-associated pneumonia (VAP), where relapse or delayed clearance frequently occurs in the absence of stable genetic resistance [174,175,176]. In these settings, persistence has been linked to biofilm-associated tolerance, metabolic reprogramming, and phenotypic diversification rather than to fixed resistance mutations.

Similarly, chronic and relapsing *S. aureus* infections are characterized by extensive phenotypic heterogeneity, including SCVs, altered metabolic states, and regulatory reprogramming that promote intracellular survival and antibiotic tolerance [177,178,179]. Longitudinal studies in CF and device-associated infections demonstrate that *S. aureus* populations adapt toward a persistent, colonizing lifestyle marked by reduced virulence gene expression and reversible phenotype switching, rather than by uniform acquisition of resistance determinants.

Plasticity-aware therapeutic strategies may exploit this vulnerability. For example, aPDI or aBL can be applied as adjuvant treatments to disrupt biofilm structure, damage stress-buffering systems, and transiently resensitize tolerant populations to antibiotics. Such approaches have been shown to enhance antibiotic efficacy in biofilm-forming pathogens, including *P. aeruginosa*, *S. aureus*, *Acinetobacter baumannii*, and enterococci, without promoting classical resistance [138,180,181,182,183]. In this context, light-based therapies act not as standalone antimicrobials but as plasticity-disrupting adjuvants that collapse transient survival states.

At the diagnostic level, integrating plasticity-aware readouts into clinical microbiology workflows represents a realistic near-term goal. In particular, incorporating measures that capture delayed killing or post-treatment recovery may help distinguish phenotypic tolerance from stable resistance. Such adaptations would be especially valuable in chronic and biofilm-associated infections, where treatment failure often reflects plastic survival rather than fixed resistance mechanisms.

### 6.5. Implications for Evolutionary Rescue Under Antimicrobial Stress

The evolutionary implications of plasticity remain insufficiently understood. Plastic responses can facilitate evolutionary rescue by buffering mortality and prolonging population persistence until adaptive mutations arise [43,159]. Conversely, priming and rapidly deployed stress responses may suppress mutagenesis and transiently stabilize phenotypes, whereas delayed responses may increase the likelihood of bursts of mutation [74,75,154]. Determining when plasticity buffers genetic change and when it promotes mutagenesis will be essential for predicting evolutionary trajectories under antimicrobial pressure. Resolving this balance will be critical for anticipating when antimicrobial interventions suppress evolutionary change and when they inadvertently promote adaptive diversification.

## 7. Materials and Methods

### Literature Search Strategy

Relevant literature was identified through systematic searches of Google Scholar, PubMed, and Scopus using combinations of keywords including phenotypic plasticity, bacterial tolerance, persistence, resilience, oxidative stress, reactive oxygen species, antimicrobial blue light, and antimicrobial photodynamic inactivation. Additional references were retrieved through citation chaining and expert knowledge of the field. All cited studies were manually verified by the author to ensure accuracy, relevance, and consistency with the conceptual framework of this review.

## 8. Conclusions

The dominant paradigm in AMR research has centered on genetic mutations and HGT. While these mechanisms are undeniably central, they represent only part of the adaptive repertoire available to bacteria. Increasingly, it has become clear that phenotypic plasticity, the capacity of cells to flexibly adjust morphology, metabolism, community organization, and regulatory states in response to stress, constitutes a fundamental yet underappreciated determinant of microbial survival.

Plasticity gives rise to diverse non-genetic outcomes, including tolerance, persistence, and resilience, enabling bacterial populations to withstand otherwise lethal treatments without measurable changes in MIC values. These transient states complicate antimicrobial diagnostics and contribute to the chronicity and recalcitrance of infections. At the same time, they offer critical insights into bacterial survival strategies that extend beyond classical resistance paradigms. Oxidative and photodynamic stresses provide particularly instructive models for studying phenotypic plasticity. They reveal that bacterial survival often depends not on fixed genetic traits, but on dynamic phenotypic states unfolding across individual cells and microbial communities.

Importantly, phenotypic plasticity should not be viewed solely as a therapeutic obstacle. It also presents exploitable vulnerabilities. Light-based antimicrobial strategies can destabilize protective phenotypes, inactivate resistance-associated enzymes, and synergize with antibiotics, thereby turning flexibility itself into a therapeutic vulnerability. Recognizing this dual nature of plasticity, as both a challenge and an opportunity, opens new avenues for interventions that target transient survival states before they stabilize into heritable resistance.

Moving beyond a resistance-centric framework requires embracing phenotypic plasticity as a core principle of microbial adaptation. Integrating plasticity into the chemical biology of AMR sharpens our understanding of infection dynamics, refines diagnostic concepts, and informs the rational design of next-generation antimicrobial strategies. In doing so, it provides not only a richer conceptual framework but also a pragmatic foundation for addressing one of the most pressing global health challenges.

## Figures and Tables

**Table 1:** Operational framework distinguishing bacterial survival strategies beyond genetic resistance.

Concept	Genetic Basis	Reversibility	Population Structure/Response	Typical Timescale	Key Features	References
**Genetic** **resistance**	Stable mutations or horizontal gene transfer	No	Entire population resistant	Long-term	Increased MIC; maintained without stress	[1,3,4]
**Tolerance**	Non-resistance-conferring genetic background and/or regulatory state	Yes	Population-level delayed killing dynamics	Short–medium (can persist across generations)	Reduced killing rate without MIC change	[4,5]
**Persistence**	Non-genetic, stochastic	Yes	Small dormant subpopulation	Transient, recurrent	Rare cells survive lethal stress; regrow upon stress removal	[8,23]
**Resilience**	Non-genetic, population-level	Yes	Post-exposure population response	Post-stress recovery phase	Capacity to regrow after treatment rather than survive exposure	[20,21,24]
**Phenotypic plasticity**	Regulatory, metabolic, physiological	Yes	Heterogeneous, dynamic	Context- dependent	Framework encompassing tolerance, persistence and resilience	[9,25,26,27]

**Table 2:** Mechanisms of phenotypic plasticity in bacterial responses to antibiotics, oxidative stress, and photodynamic inactivation. The table highlights representative phenotypes, their triggers, and implications for bacterial survival and adaptation.

Level/Mechanism	Phenotypic Manifestation	Trigger	Implications	References
**Morphological**	Filamentation	β-lactams, quinolones, ROS	SOS-induced elongation; transient tolerance to ROS and immune attack	[9,10,160]
	SCVs	Aminoglycosides, ROS, aPDI	Slow growth, metabolic rewiring, persistence in host tissues	[41,85,132]
	L-forms	β-lactams, oxidative stress	Cell wall-deficient states enable transient survival under cell wall stress	[9,88]
**Metabolic**	PPP/TCA rewiring → ↑ NADPH	Antibiotics, ROS	Enhanced antioxidant defenses	[59]
	Priming responses	Sublethal H_2_O_2_	Induction of protective programs; reduced killing	[74]
	Adaptation delay → mutagenesis	Sudden H_2_O_2_	Transient window of increased mutagenesis	[75]
**Envelope & biofilm**	Envelope stress remodeling (RpoS, porins, efflux)	Antibiotics, ROS, aBL	Altered permeability and efflux; transient tolerance	[41,98,161]
	Biofilm heterogeneity	Antibiotics, ROS, aPDI	Microenvironments generate tolerant/persister subpopulations	[16,32,33,162,163]
	Biofilm–planktonic switching	Environmental fluctuations	Plastic transitions favored under variable conditions	[18]
**Regulatory**	Rap/Phr signaling	Stress, biofilm (*Bacillus* spp.)	Context-dependent control of sporulation, motility, competence	[109]
	HRT	Mobility of non-coding regulatory regions	Network rewiring without coding changes	[110]
**Mobile elements**	Plasmid-dependent plasticity	Antibiotics, environmental stress	Fitness trade-offs; modulation of stress responses	[113,114]

Abbreviations: aBL, antimicrobial blue light; aPDI, antimicrobial photodynamic inactivation; ROS, reactive oxygen species; PPP, pentose phosphate pathway; TCA, tricarboxylic acid cycle; SCV, small-colony variant; HRT, horizontal regulatory transfer. The arrow (↑) indicates an increase.

## Data Availability

No new data were created or analyzed in this study. Data sharing is not applicable to this article.

## Abbreviations

The following abbreviations are used in this manuscript:

aBL	antimicrobial Blue Light
AMR	antimicrobial resistance
aPDI	antimicrobial Photodynamic Inactivation
CF	cystic fibrosis
EPS	extracellular polymeric substances
GRN	gene regulatory network
HRT	horizontal regulatory transfer
ICEs	integrative and conjugative elements
LLPS	liquid–liquid phase separation (LLPS)
MDK	minimum duration of killing
MGEs	mobile genetic elements
MIC	minimum inhibitory concentration
NADPH	nicotinamide adenine dinucleotide phosphate (reduced form)
(p)ppGpp	guanosine tetraphosphate and guanosine pentaphosphate
RB	Rose Bengal
ROS	reactive oxygen species
SCVs	small colony variants
VAP	ventilator-associated pneumonia
VBNC	viable but non-culturable
